# Hepatoprotective action of various partitions of methanol extract of *Bauhinia purpurea* leaves against paracetamol-induced liver toxicity: involvement of the antioxidant mechanisms

**DOI:** 10.1186/s12906-016-1110-4

**Published:** 2016-06-11

**Authors:** Zainul Amiruddin Zakaria, Farhana Yahya, Siti Syariah Mamat, Nur Diyana Mahmood, Nurhafizah Mohtarrudin, Muhammad Taher, Siti Selina Abdul Hamid, Lay Kek Teh, Mohd. Zaki Salleh

**Affiliations:** Department of Biomedical Science, Faculty of Medicine and Health Sciences, Universiti Putra Malaysia, 43400 Serdang, Selangor Malaysia; Pharmacogenomics Centre (PROMISE), Faculty of Pharmacy, Universiti TeknologiMARA, 42300 Puncak Alam, Selangor Malaysia; Department of Pathology, Faculty of Medicine & Health Sciences, Universiti Putra Malaysia, 43400 Serdang, Selangor Malaysia; Department of Pharmaceutical Technology, Kulliyyah of Pharmacy, International Islamic University Malaysia, Jalan Istana, Bandar Indera Mahkota, 25200 Kuantan, Pahang Malaysia; Medical Technology Division, Malaysian Nuclear Agency, Bangi, 43000 Kajang, Selangor Malaysia

**Keywords:** *Bauhinia purpurea*, Fabaceae, Hepatoprotection, Antioxidant, Flavonoids, Synergistic action

## Abstract

**Background:**

Methanol extract of *Bauhinia purpurea* L. (family Fabaceae) (MEBP) possesses high antioxidant and anti-inflammatory activities and recently reported to exert hepatoprotection against paracetamol (PCM)-induced liver injury in rats. In an attempt to identify the hepatoprotective bioactive compounds in MEBP, the extract was prepared in different partitions and subjected to the PCM-induced liver injury model in rats.

**Methods:**

Dried MEBP was partitioned successively to obtain petroleum ether (PEBP), ethylacetate (EABP) and aqueous (AQBP) partitions, respectively. All partitions were subjected to in vitro antioxidant (i.e. total phenolic content (TPC), 2,2-diphenyl-1-picrylhydrazyl (DPPH)- and superoxide-radicals scavenging assay, and oxygen radical absorbance capacity (ORAC) assay) and anti-inflammatory (i.e. lipooxygenase (LOX) and xanthine oxidase (XO) assay) analysis. The partitions, prepared in the dose range of 50, 250 and 500 mg/kg, together with a vehicle (10 % DMSO) and standard drug (200 mg/kg silymarin) were administered orally for 7 consecutive days prior to subjection to the 3 mg/kg PCM-induced liver injury model in rats. Following the hepatic injury induction, blood samples and liver were collected for the respective biochemical parameter and histopathological studies. Body weight changes and liver weight were also recorded. The partitions were also subjected to the phytochemical screening and HPLC analysis.

**Results:**

Of all partitions, EABP possessed high TPC value and demonstrated remarkable antioxidant activity when assessed using the DPPH- and superoxide-radical scavenging assay, as well as ORAC assay, which was followed by AQBP and PEBP. All partitions also showed low anti-inflammatory activity via the LOX and XO pathways. In the hepatoprotective study, the effectiveness of the partitions is in the order of EABP>AQBP>PEBP, which is supported by the microscopic analysis and histopathological scoring. In the biochemical analysis, EABP also exerted the most effective effect by reducing the serum level of alanine transaminase (ALT) and aspartate transaminase (AST) at all doses tested in comparison to the other partitions. Phytochemical screening and HPLC analysis suggested the presence of: flavonoids, condensed tannins and triterpenes in EABP; flavonoids, condensed tannins and saponins in PEBP and; only saponins in AQBP.

**Conclusion:**

EABP demonstrates the most effective hepatoprotection against PCM-induced liver injury in rats. This observation could be attributed to its remarkable antioxidant activity and the presence of flavonoids that might probably act synergistically with other biocompounds to cause the hepatoprotection.

## Background

One of the most widely used drugs prescribed for liver treatment to counteract poisoning by the well-known over-the-counter analgesic (i.e.paracetamol; PCM) is N-Acetylcysteine (NAC). The use of NAC as an antioxidant to effectively replenish glutathione (GSH) intracellular stores in the circulatory system has demonstrated encouraging results in animal models of reperfusion injuries and ischemia, and it has been consumed by humans for several years [1]. However, the effectiveness of NAC has been surpassed by several reports on its adverse effects on health [2]. Due to this problem, there is still a need to search for alternative agents for the treatment of liver ailments with less or, possibly, no side effects, cheaper and widely available. Plants have been one of the good sources of new bioactive compounds [3] and, interestingly, Malaysia, with its rich flora and fauna, have been one of the countries that supported researches related to natural product-based drug discoveries [4].

In an attempt to contribute to the field of natural product-related drug discoveries, we have exploited several plants wildly cultivated in Malaysia but considered previously to be neglected and underutilized plants [5]. One of the plants is *Bauhinia purpurea* L., which belongs to the family Fabaceae. Locally known as ‘*Tapak Kerbau*’, this plant is native to Malaysia but no traditional medicinal use has been recorded among the Malay tribes. However, *B. purpurea* has medicinal values to the peoples in the Indian region wherein it is used to treat ulcer wounds, stomach tumors, fever, diarrhea and glandular swellings [6]. Through scientific studies, various pharmacological potentials of *B. purpurea* have been reported such as antioxidant [7, 8, 9], antiulcer [10, 11], anti-inflammatory [7, 12, 13], antinociceptive, antipyretic [13, 14, 15], antiproliferative [9], antimicrobial [16], and wound healing [17]. In addition to these, we have recently reported on the hepatoprotective activity of methanol extract of *B. purpurea* leaves against the carbon tetrachloride (CCl_4_)-induced liver toxicity model [6]. The mechanisms of hepatotoxicity induced by CCl_4_ differ from other agents such as paracetamol (PCM) [18]. Based on previous reports on the overuse of PCM and its adverse side effects [2] and that its mechanism of hepatotoxicity differs from CCl_4_, there is a need to find alternative cure to PCM-induced hepatotoxicity. Therefore, the present study was proposed to study the partitions of methanol extract of *B. purpurea* leaves (MEBP) potentials to attenuate the PCM-induced liver toxicity in rats.

## Methods

### Chemicals

Methanol, petroleum ether, and ethyl acetate (Fisher Scientific UK, Loughborough), Paracetamol (PCM) and silymarin (*Sigma*-Aldrich, USA) were used in the present study. All other chemicals and reagents used were of analytical grade.

### Collection of plant material

The leaves of *B. purpurea* were collected around Universiti Putra Malaysia (UPM), Serdang, Selangor, Malaysia. A voucher specimen (SK 1985/11) was identified by comparison with specimens available at the Herbarium of the Laboratory of Natural Products, Institute of Bioscience, UPM. The leaves were dried under shade for 7 days at room temperature, segregated, and pulverized by mechanical grinder to form a coarse powder.

### Preparation of methanol extract of B. purpurea (MEBP)

The coarse powder of *B. purpurea* (1 kg) was macerated in 20 L methanol (ratio of 1:20 (w/v) was used) for 72 h. The supernatant was collected and filtered sequentially using cloth filter, cotton wool and Whatman No. 1 filter paper followed by evaporation (Buchi Rotavapor® R210/215, Switzerland) under reduced pressure (204 mbar) and controlled temperature (40 °C). The plant residue was collected and subjected to the similar extraction process for another two times.

### Preparation of petroleum ether, ethyl acetate and aqueous partitions from MEBP

Petroleum ether, ethyl acetate, and aqueous extraction of MEBP were achieved by the standard solvent partitioning methods described by Balan et al. [19] with slight modifications. MEBP (2 g) was dissolved in 100 mL methanol and 200 mL distilled water. The solution (suspension) was partitioned accordingly as 3 × 700 mL petroleum ether and 3 × 700 mL ethyl acetate, successively, yielding the aqueous partition at the end of the process. The 2-phase immiscible liquid solutions, obtained during each of the petroleum ether or ethyl acetate partitioning, were separated after 30 min. The solvent was then evaporated under reduced pressure (204 Mbar) and controlled temperature (40 °C) using a vacuum rotary evaporator (Buchi Rotavapor® R210/215, Switzerland); the aqueous extract was then subjected to the freeze-drying process.

### Antioxidant activity of PEBP, EABP and AQBP

#### Total phenolic content

Determination of total phenolic content (TPC) was performed using Folin-Ciocalteu reagent according to the method of Singleton and Rossi [20] with slight modifications. Briefly, 1.0 mg of the respective partition was extracted for 2 h with 1.0 mL 80 % methanol containing 1.0 % hydrochloric acid and 1.0 % distilled water at room temperature on a shaker at 200 rpm. The mixture was centrifuged at 6000 rpm for 15 min and the supernatant was decanted into vials. The supernatant was used for the determination of TPC. The supernatant extract (200 μL) was mixed with 400 μL Folin-Ciocalteu reagent (0.1 mL/0.9 mL) and allowed to stand at room temperature for 5 min. Then, 400 μL sodium bicarbonate (60.0 mg/mL) solution was added, and the mixture was allowed to stand at room temperature for 90 min. Absorbance was measured at 725 nm. A calibration curve was generated by using the optical density (OD) of the gallic acid (GA) standard, and the levels in the samples were expressed as GA equivalent (GAE)-TPC mg/100 g.

### 2,2-diphenyl-1-picrylhydrazyl (DPPH) radical scavenging assay

The antioxidant reducing activity of each partition on DPPH radicals was estimated according to the method of Blois [21] with modifications involving the use of a high-throughput microplate system. Sample (50 μL of 1.0 mg/mL) was added to 50 μL DPPH (FG: 384.32) (1 mM in ethanolic solution) and 150 μL ethanol (absolute) in a 96-well microtiter plate in triplicate. The plate was shaken (15 s, 500 rpm) and left to stand at room temperature for 30 min. The absorbance of the resulting solution was measured spectrophotometrically at 520 nm. Data were analyzed using GraphPad PRISM® V5.01 (GraphPad Software, La Jolla, CA, USA). The IC_50_ was determined from an overdose–response curve. The IC_50_ of a sample can be determined by constructing a dose–response curve and examining the effect of different concentrations, namely 3.13, 6.25, 25, 50, and 200 μg/mL.

### Xanthine/xanthine oxidase (X/XOD) *superoxide scavenging assay*

The assay was performed according to the method of Subramaniam et al. [22] with slight modifications. Nitroblue tetrazolium (NBT) solution (100 mL of 4.1 mM/L) was prepared by adding 3.15 g Tris–HCl, 0.1 g MgCl_2_, 15.0 mg 5-bromo-4-chloro-3-indolyl phosphate, and 34.0 mg 4-NBT chloride to 100 mL distilled water. The reaction mixture (100 mL) was prepared by dissolving 0.53 g Na_2_CO_3_ (pH 10.2), 4.0 mg EDTA, and 2.0 mg xanthine in 0.025 mM NBT solution. The mixture was kept refrigerated at 4 °C. For the negative control group, approximately 999 uL of the reaction mixture was transferred into a microcuvette and placed in a cell holder of a spectrophotometer maintained at 25 °C. This was followed by the addition of 1x10^−3^ U/ml of xanthine oxidase (XOD) to initiate the generation of superoxide formation. The optical density (OD) measurements were taken at 560 nm for 120 s using a Lambda 2S spectrophotometer. For the positive control group, approximately 979 uL of the reaction mixture was transferred into a microcuvette and placed in a 25 °C cell holder of a spectrophotometer. Then 1.16U/mL of superoxide dismutase (SOD) was added into the reaction mixture and thoroughly mixed. XOD (1x10-3 U/mL) was then added to start the generation of superoxide formation. OD was taken at 560 nm for 120 s at intervals of 10 s. For the treatment group, approximately 5 uL of the respective 200 ug/mL PEBP, EABP or AQBP, were dissolved in approximately 994 uL of the reaction mixture that was placed in a cell holder of a spectrophotometer maintained at 25 °C. XOD (1x10^−3^ U/ml) was then added and after thoroughly mixing, similarly measured for the XOD and SOD curves.

### Oxygen radical absorbance capacity (ORAC) test

The oxygen radical absorbance capacity (ORAC) test was carried out according to Huang et al. [23] with slight modifications. 2,2’-Azobis(2-methylpropionamidine) dihydrochloride (AAPH) (0.65 g) was dissolved in 10 mL 75 mM phosphate buffer (pH 7.4) to a concentration of 240 mM, and was freshly made. A fluorescein stock solution (1 mM) was prepared in 75 mM phosphate buffer (pH 7.4), wrapped in foil, and stored at 5 °C. Immediately before use, the stock solution was diluted with 75 mM phosphate buffer at 1:100,000. The diluted sodium fluorescein was prepared daily as a fresh solution. The sodium fluorescein solution (150 mL) was placed in the interior experimental wells. The blanks contained 25 mL Trolox dilution. The sample wells contained 25 mL sample. The plate was then incubated for 10 min at 37 °C for equilibration. BMG Omega Fluostar Fluorescent Spectrophotometer (BMG LABTECH, Germany) with injector using an emission filter of 528-nm bandpass and excitation filter of 485-nm bandpass was used. The plate reader was analyzed using MARS Data Analysis software (BMG LABTECH, Germany). AAPH solution (25 mL, 240 mM) was added using the injector of the microplate reader to a final volume of 200 mL to start the reaction. The 25 mL AAPH solution was then mixed by shaking for 50 s at maximum intensity. The fluorescence was observed kinetically, with data recorded every minute. The reading of the fluorescence of each well was measured every 60 s. ORAC values were analyzed using MARS Data Analysis Reduction software (BMG LABTECH, Germany). All assays were performed in three independent replicates.

### Anti-Inflammatory assays

#### Lipoxygenase assay

Lipoxygenase (LOX)-inhibiting activity was detected using the spectrophotometric method as described by Malik et al. [24]. Sodium phosphate buffer (160 mL, 0.1 M, pH 8.0), 20 mL soybean LOX solution, and 10 mL of each partition were mixed and incubated at 25 °C for 10 min. The reactions were initiated by the addition of 10 mL substrate in the form of sodium linoleic acid solution. The enzymatic conversion of linoleic acid to form (9Z, 11E)-(13S)-13-hydroperoxyoctadeca-9,11-dienoate causes the change in absorbance measured at 234 nm over a period of 6 min. The reference standard, nordihydroguaiaretic acid (NDGA) and partitions were dissolved in methanol. All reactions were performed in triplicate in a 96-well microplate.

#### Xanthine oxidase assay

The procedure was carried out according to the spectrophotometric method described by Noro et al. [25] to measure the activity of the partitions in xanthine oxidase (XO) inhibition. XO solution (10 μL), 130 μL potassium phosphate buffer (0.05 M, pH 7.5), and 10 μL of test extracts were mixed together and incubated at 25 °C for 10 min. The reaction started after the addition of 100 μL substrate in the form of xanthine solution. The conversion from xanthine to hydrogen peroxide and uric acid was measured at 295 nm. The reference standard, Allopurinol and tested extracts were dissolved in DMSO. All reactions were completed in a 96-well UV microplate in triplicate.

### Experimental animals

The study protocol of the present study was approved by the Animal Care and Use Committee (ACUC), Faculty of Medicine and Health Sciences, UPM (Ethical approval no.: UPM/FPSK/PADS/BR-UUH/00450). Male Sprague Dawley rats (180–200 g; 8–10 weeks old) were obtained from the Veterinary Animal Unit, Faculty of Veterinary Medicine, Universiti Putra Malaysia (UPM), Malaysia and were house at room temperature (27 ± 2 °C; 70 – 80 % humidity; 12 h light/darkness cycle) in the Animal Holding Unit (UPM). They were supplied with food and water ad libitum from the beginning of the experiments. The rats were handled in accordance with current UPM guidelines for the care of laboratory animals and the ethical guidelines for investigations of experimental pain in conscious animals. All experiments were conducted between 09.30 and 18.30 h to minimize the effects of environmental changes.

### Hepatoprotective assay

The in vivo hepatoprotective activity of PEBP, EABP and AQBP were determined using PCM-induced hepatotoxicity in rats. As described in Table 1, the groups were assigned and administered with the respective test solutions. The procedures for PCM were carried out according to Porchezhian and Ansari [26]. The animals were fasted for 48 h prior to the experiment under standard laboratory conditions. After 48 h, each group of rats received the respective dose of test solution orally once daily for 7 consecutive days. The administration of PCM (3 mg/kg, p.o) was performed 3 h after the last dose of each partition administration on day 7, except for group I, which received only 10 % dimethyl sulfoxide (DMSO). Forty-eight hours after the hepatic injury induction, the animals were anesthetized using diethyl ether and blood samples were obtained via cardiac puncture using a sterile disposable syringe for biochemical parameter study. The body weights of the animals were measured before they were sacrificed. Subsequently, the rats were sacrificed and then the livers were harvested, washed in normal saline, blotted with filter paper, and weighed. Gross examination was conducted to examine for any abnormalities in the organ. The livers of all animals were subsequently subjected to antioxidant assays and histopathological examination.

**Table 1:** PCM-induced hepatotoxicity treatment groups of the extracts

Group	Extracts	Doses of extracts
I.		Normal control: 10 % DMSO, p.o
II.		Negative control: 10 % DMSO, p.o
Ill.		Positive control: 200 mg/kg silymarin, p.o
IV.	PEBP	50 mg/kg PEBP, p.o
V.		250 mg/kg PEBP, p.o
VI.		500 mg/kg PEBP, p.o
VlI.	EABP	50 mg/kg EABP, p.o
VllI.		250 mg/kg EABP, p.o
IX.		500 mg/kg EABP, p.o
X.	AQBP	50 mg/kg AQBP, p.o
XI.		250 mg/kg AQBP, p.o
XlI.		500 mg/kg AQBP, p.o

### Biochemical studies

The plasma samples were collected for measuring liver markers. Biochemical parameters were assayed according to standard methods. Alanine transaminase (ALT), alkaline phosphatase (ALP), aspartate transaminase (AST), total protein (TP), and lactate dehydrogenase (LDH) levels were measured using a Hitachi 902 Automatic Chemical Analyser, USA.

### Histopathology

The liver tissue was dissected out and fixed in 10 % formalin, dehydrated in graded ethanol (50–100 %), cleared in xylene, and embedded in paraffin wax. The sections, which were 5–6-μm thick, were then prepared using a rotary microtome (Leica RM2125 RTS, Singapore) and stained with hematoxylin and eosin dye for microscopic observation of histopathological changes in the liver. Liver sections were then scored and evaluated according to the severity of the hepatic injury as described by El-Beshbishy et al. [27].

### Phytochemical screening of PEBP, EABP and AQBP

Phytochemical screening of PEBP, EABP and AQBP was performed according to standard screening tests and conventional protocols as described in detail by Kamarolzaman et al. [28]. The analysis of phytoconstituents in those partitions was performed to detect alkaloids, flavonoids, triterpenes, tannins, saponins and steroids.

### HPLC Profiling of PEBP, EABP and AQBP

The HPLC analysis of PEBP, EABP and AQBP was performed according to a previous report [19]. Approximately, 10 mg of the respective partition was suspended in 1 ml methanol and then filtered through the membrane filter with a pore size of 0.45 μm. A Waters Delta 600 with 600 Controller and Waters 2996 Photodiode Array (Milford, MA, USA) equipped with an autosampler, online degasser and column heater was used to analyze the filtered sample. Data was evaluated and processed using the installed Millenium 32 Software (Waters Product). The filtered samples were separated at 27 °C on a minibore Phenomenex Luna 5 μm C18 column (dimensions 250 x 4.60 mm) using a one-step linear gradient. Two solvents denoted as A, which is 0.1 % aqueous formic acid, and B, which is acetonitrile, were used to elute the constituents. Initial conditions were set at 95 % A and 5 % B with a linear gradient reaching 25 % B at t = 12 min. This condition was maintained for 8 min before B was reduced back to 15 % at t = 22 min for another 8 min (t = 30 min). At t = 35 min, the programme was returned to the initial solvent composition. The flow rate used was 1.0 ml/min and the injection volume was 10 μl. The column oven was set at 27 °C and the eluent was monitored at 210, 254, 280, 300, 330 and 366 nm. The retention times, peak areas and UV spectra of the major peaks were analyzed.

### Statistical analysis

Data obtained are presented in Mean ± Standard Error of Mean (SEM). The data were analyzed using the one-way analysis of variance (ANOVA) and the differences between all partition-treated group were determined using the Tukeys post-hoc test with *P* < 0.05 as the limit of significance.

## Result

### In vitro antioxidant activity of all fractions

#### Total phenolic content (TPC) value

Table 2 shows the TPC value of PEBP, EABP, and AQBP at the concentration of 200 μg/mL. Based on the standard protocol, compounds with a TPC value that is higher than 1000 mg GAE/100 g can be considered to have a high TPC value. Hence, all partitions, which possessed the TPC value between 100–300 mg/100 g GAE can be considered as to have low TPC value in the sequence of EABP > AQBP > PEBP.

**Table 2:** Total phenolic content of different extracts

Sample	Total phenolic content (TPC) mg/100 g GAE
PEBP	104.83 ± 1.64
EABP	270.27 ± 39.06
AQBP	132.08 ± 15.88

Total phenolic content are expressed as GAE (gallic acid equivalent)

All values are expressed as mean ± SEM

#### DPPH- and superoxide- radical scavenging activities and ORAC value

PEBP and AQBP, at the concentration of 200 μg/mL, demonstrated a very low antioxidant activity when assessed using the DPPH radical scavenging assay with the recorded percentage of antioxidant of 17.1 and 19.4 %, respectively. On the other hand, 200 μg/mL EABP showed the highest antioxidant activity (64.6 %) among the partitions tested, but, overall EABP was considered to have a moderate antioxidant activity.

For the superoxide scavenging activity, EABP also showed the highest percentage of scavenging activity, followed by PEBP, and AQBP (Table 3).

**Table 3:** Superoxide scavenging activity of different extracts

Sample	Superoxide scavenging (%)
PEBP	62.20 ± 3.55
EABP	88.30 ± 5.25
AQBP	44.7 ± 3.15

The data are expressed as the percentage of free radical scavenging activity

All values are expressed as mean ± SEM

Note: H, high (71–100 %); M, moderate (41–70 %); L, low (0–40 %); NA, not active

In the ORAC assay, AQBP had the highest ORAC value, followed by EABP and PEBP (Table 4).

**Table 4:** Oxygen radical absorbance capacity of different extracts

Sample	ORAC value (TE/100 g)
PEBP	48,000
EABP	168,000
AQBP	172,000

The ORAC values are expressed in micromoles of Trolox Equivalents per 100 g of samples

### In vitro anti-inflammatory activity

#### LOX and XO activities

In both assays, all partitions caused weak inhibition of LOX and XO activities at the concentration of 200 μg/mL with the highest percentage of inhibition recorded only at 15 and 17 %, respectively (Table 5).

**Table 5:** Effect of extracts on the anti-inflammatory mediators using the in vitro lipoxygenase and xantine oxidase assays

Sample	Lipoxygenase (%)	Xanthine oxidase (%)
NDGA (reference standard of LOX)	99.86 ± 0.14	-
Allopurinol (reference standard of XO)	-	97.58 ± 0.32
PEBP	NA	8.57 ± 0.86
EABP	15.05 ± 2.33	17.05 ± 3.14
AQBP	9.65 ± 1.10	1.65 ± 0.84

All values are expressed as mean ± SEM

Note: H, high (71–100 %); M, moderate (41–70 %); L, low (0–40 %); NA, not active

#### In vivo hepatoprotective activity

##### Effect of PEBP, EABP and AQBP on the rats’ liver/body weight ratio after induction with PCM

A significant (*P* < 0.05) augmentation of body weight was observed in the group that received PCM following pre-treatment with 10 % DMSO when compared to the normal control group. Only EABP and AQBP, at the concentration of 250 and 500 mg/kg, caused significant (*P* < 0.05) decreased in body weight (Table 6). With regard to the liver/body weight ratio, only EABP, at the dose of 500 mg/kg, caused significant (*P* < 0.05) decrease in the liver/body weight ratio. In comparison, 200 mg/kg silymarin also caused a significant (*P* < 0.05) reduction in the body weight and liver/body weight ratio in comparison to the hepatotoxic group (Table 6).

**Table 6:** Effect of PEBP, EABP and AQBP on percentage change of liver weight in PCM- treated rats

Treatment	Dose (mg/kg)	Change of body weight (%)	Liver/body weight ratio (g/100 g)
Normal control	-	5.14 ± 0.43	2.86 ± 0.05
10 % DMSO + PCM		17.58 ± 2.10^a^	4.58 ± 0.33^a^
Silymarin + PCM	200	3.11 ± 0.67^b^	3.59 ± 0.10^b^
PEBP + PCM	50	14.20 ± 1.64	4.24 ± 0.14
	250	14.69 ± 2.60	4.20 ± 0.32
	500	15.84 ± 1.34	3.82 ± 0.05^a^
EABP + PCM	50	15.20 ± 1.07	4.53 ± 0.32
	250	**3.72 ± 1.10** ^**b**^	4.62 ± 0.17
	500	**4.01 ± 0.98** ^**b**^	3.95 ± 0.19^a^
AQBP + PCM	50	**9.78 ± 1.13** ^**b**^	4.16 ± 0.11
	250	**8.26 ± 0.91** ^**b**^	4.39 ± 0.21
	500	14.26 ± 1.76	4.18 ± 0.25

Values are expressed as means ± S.E.M. of six replicates

^a^ Significant different (*p* < 0.05) when compared to the normal control group ((10 % DMSO + dH_2_O)-treated) of the respective column

^b^ Significant different (*p* < 0.05) when compared to the negative control group ((10 % DMSO + PCM)-treated) of the respective column

**Table 7:** Histopathological evaluation of the effects of PEBP, EABP and AQBP against PCM-induced hepatic injury in rats

Treatment	Dose (mg/kg)	Steatosis	Necrosis	Inflammation	Haemorrhage
Normal control	-	-	-	-	-
10 % DMSO + PCM		-	+++	++	+
Silymarin + PCM	200	-	+	+	-
PEBP + PCM	50	-	+++	++	-
	250	-	++	++	-
	500	-	++	++	-
EABP + PCM	50	-	++	++	-
	250	-	+	+	-
	500	-	+	+	-
AQBP + PCM	50	-	++	++	-
	250	-	+	++	-
	500	-	+	++	-

The severity of various features of hepatic injury was evaluated based on those following scoring scheme: − normal, + mild effect, ++ moderate effect, +++ severe effect

### Histopathological study

**Fig. 1:**
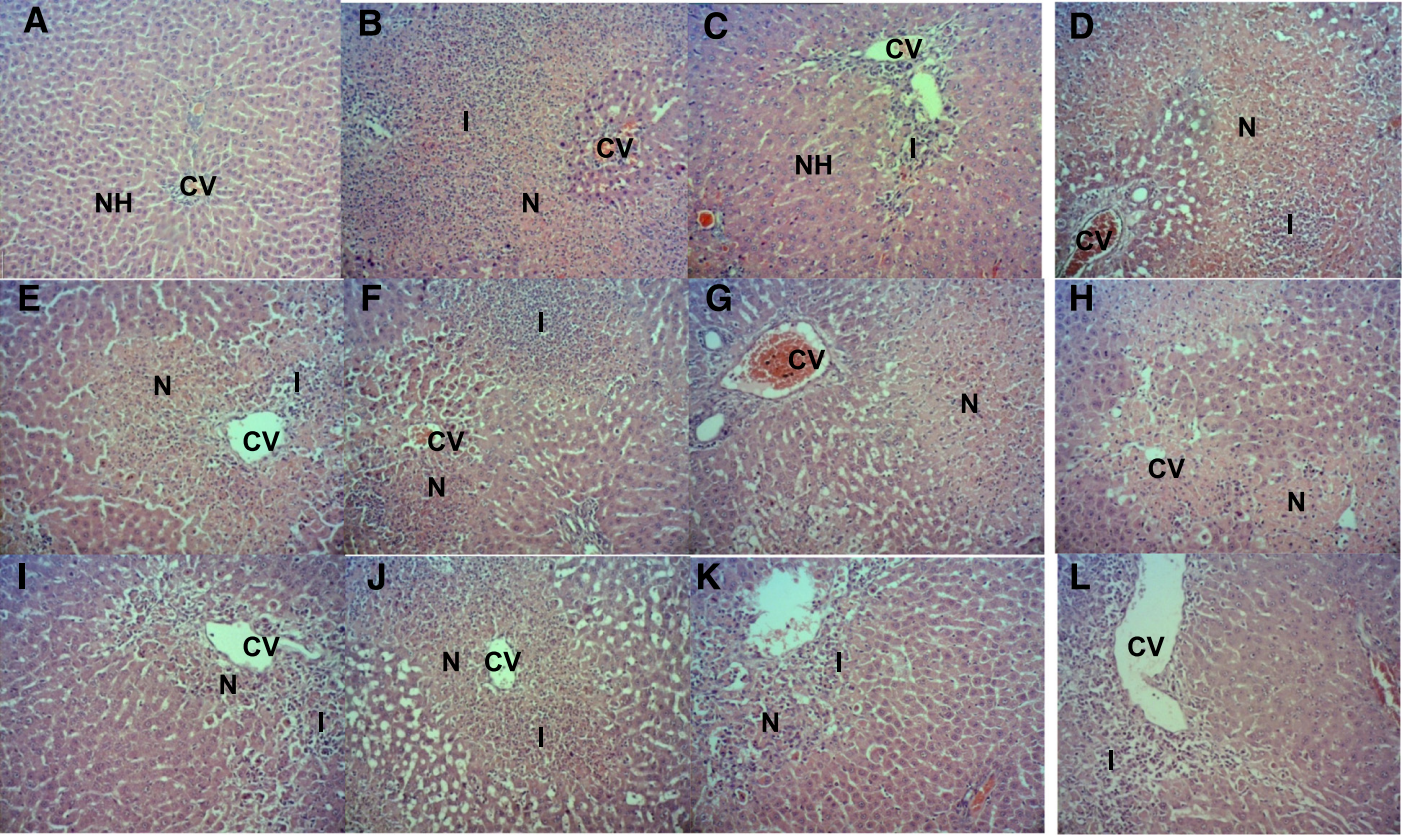
**a** Normal liver parenchyma, **b** Section of liver tissue treated with 3 g/kg PCM (p.o) showing massive necrosis, and inflammation. **c** Section of liver tissue pre-treated with 200 mg/kg silymarin followed by PCM showing preservation of normal hepatocytes. **d** Section of liver tissue pre-treated with 50 mg/kg PEBP followed by PCM showing massive tissue necrosis and mild inflammation. **e** Section of liver tissue pre-treated with 250 mg/kg PEBP followed by PCM showing moderate necrosis and mild inflammation. **f** Section of liver tissue pre-treated with 500 mg/kg PEBP followed by PCM showing moderate necrosis and inflammation. **g** Section of liver tissue pre-treated with 50 mg/kg EABP followed by PCM showing moderate necrosis. **h** Section of liver tissue pre-treated with 250 mg/kg EABP followed by PCM showing mild necrosis. **i** Section of liver tissue pre-treated with 500 mg/kg EABP followed by PCM showing mild inflammation and necrosis. **j** Section of liver tissue pre-treated with 50 mg/kg AQBP followed by PCM showing moderate inflammation and necrosis. **k** Section of liver tissue pre-treated with 250 mg/kg AQBP followed by PCM showing mild necrosis and inflammation. **l** Section of liver tissue pre-treated with 500 mg/kg AQBP followed by PCM showing mild inflammation. (H & E, X100). CV) central vein. N) necrosis. I) inflammation

Histopathological study was performed on the liver to observe any deformities in the structure. The group pre-treated with 10 % DMSO (normal) without PCM intoxication presented normal hepatic cells with well-preserved cell structure (Fig. 1a). The group pre-treated with 10 % DMSO followed by PCM administration had infiltration of lymphocytes and massive necrosis of the perivenular and midzonal regions (Fig. 1b). In the PEBP–pre-treated group, the pathological changes to the liver were not significantly different between groups that received various doses of PEBP, in which the liver mostly had moderate necrosis and moderate inflammation (Fig. 1d-f). Groups treated with 250 and 500 mg/kg EABP presented mild necrotic regions and mild inflammation in comparison to the group treated with 50 mg/kg EABP that showed moderate necrosis (Fig. 1g-i). Meanwhile, the group pre-treated with 50 mg/kg AQBP demonstrated moderate necrosis and inflammation that tend to improve remarkably leaving only mild inflammation at the centrilobular zone after pre-treatment with the 500 mg/kg AQBP (Fig. 1j-l). Administration of 200 mg/kg silymarin reversed the PCM-induced intoxication (Fig. 1c).

### Biochemical study

PCM administration significantly (*P* < 0.05) increased serum ALT, AST, ALP levels in the group pre-treated with 10 % DMSO when compared to the normal non–PCM-intoxicated group (Table 8). From the results obtained, only EABP significantly (*P* < 0.05) reduced the elevated serum ALT level, at all doses tested, followed by AQBP and PEBP. Meanwhile, EABP followed by PEBP and AQBP significantly (*P* < 0.05) reduced the elevated serum AST level but did not affect the serum ALP level value. In comparison, silymarin (200 mg/kg) exhibited the ability to counteract the toxic effect of PCM by significantly (*P* < 0.05) decreasing the level of all liver enzymes in comparison to the PCM-intoxicated group (Table 8).

**Table 8:** Effect of PEBP, EABP and AQBP on liver function enzymes, ALT, AST and ALP

Treatment	Dose (mg/kg)	ALT (U/L)	AST (U/L)	ALP (U/L)
Normal control	-	13.1 ± 1.0	89.6 ± 2.5	110.0 ± 6.4
10 % DMSO + PCM		1797.0 ± 141.2^a^	2970.0 ± 254.0^a^	338.8 ± 50.7^a^
Silymarin + PCM	200	517.5 ± 181.1^b^	535.5 ± 98.8^b^	201.0 ± 11.8^b^
PEBP + PCM	50	1844.0 ± 105.1	2158.0 ± 126.8^b^	371.4 ± 38.9
	250	1831.0 ± 199.0	1960.0 ± 131.1^b^	316.0 ± 30.5
	500	1219.0 ± 150.5^b^	1558.0 ± 74.7^b^	308.2 ± 20.4
EABP + PCM	50	1036 .0 ± 155.2^b^	1846.0 ± 189.9^b^	340.4 ± 58.4
	250	519.7 ± 143.4^b^	1807.0 ± 233.3^b^	320.0 ± 25.9
	500	543.9 ± 184.1^b^	895.8 ± 238.0^b^	256.0 ± 36.9
AQBP + PCM	50	1777.0 ± 119.0	2930.0 ± 151.1	338.2 ± 34.0
	250	1338.0 ± 147.2^b^	2017.0 ± 262.1^b^	329.2 ± 29.2
	500	719.3 ± 90.9^b^	1243.0 ± 190.2^b^	317.6 ± 31.0

Values are expressed as means ± S.E.M. of six replicates

^a^ Significant different (*p* < 0.05) when compared to the normal control group ((10 % DMSO + dH_2_O)-treated) of the respective column

^b^ Significant different (*p* < 0.05) when compared to the negative control group ((10 % DMSO + PCM)-treated) of the respective column

In addition, PCM-intoxicated rats demonstrated significant (*P* < 0.05) decreased in the serum level of TP but increased in the serum level of LDH when compared to the normal non–PCM-intoxicated group (Table 9). Interestingly, all partitions, at all doses tested, significantly (*P* < 0.05) reversed the toxic effect of PCM while increasing the level of TP in a dose-independent manner. Meanwhile, all partitions, at all doses tested, significantly (*P* < 0.05) decreased the serum level of LDH also in a dose-independent manner (Table 9).

**Table 9:** Effect of PEBP, EABP and AQBP on total protein (TP; g/dL) and LDH (U/L) levels

Treatment	Dose (mg/kg)	TP (g/dL)	LDH (U/L)
Normal control	-	64.1 ± 3.8	408.2 ± 60.6
10 % DMSO + PCM		50.4 ± 1.4^a^	1454.0 ± 255.0^a^
Silymarin + PCM	200	69.1 ± 2.1^b^	462.0 ± 59.8^b^
PEBP + PCM	50	77.5 ± 3.5^b^	1065.0 ± 127.3
	250	65.3 ± 1.4^b^	371.0 ± 35.7^b^
	500	76.5 ± 2.9^b^	702.4 ± 90.7^b^
EABP + PCM	50	88.1 ± 4.1^b^	765.0 ± 199.9^b^
	250	66.9 ± 0.9^b^	415.0 ± 103.0^b^
	500	83.9 ± 2.3^b^	727.8 ± 130.8^b^
AQBP + PCM	50	68.9 ± 3.8^b^	689.2 ± 148.8^b^
	250	70.4 ± 2.0^b^	905.4 ± 65.2^b^
	500	68.2 ± 1.9^b^	525.2 ± 33.9^b^

Values are expressed as means ± S.E.M. of six replicates

^a^Significant different (*p* < 0.05) when compared to the normal control group ((10 % DMSO + dH_2_O)-treated) of the respective column

^b^Significant different (*p* < 0.05) when compared to the negative control group ((dH_2_O + PCM)-treated) of the respective column

### Phytochemical screening of PEBP, EABP and AQBP

Phytochemical constituents of PEBP, EABP and AQBP are shown in Table 10. PEBP was found to contain flavonoids, condensed tannins, triterpenes and steroids; EABP was found to contain saponins, flavonoids, condensed tannins and steroids, and; AQBP was found to contain only saponins.

**Table 10:** Phytochemical constituents of PEBP, EABP and AQBP in comparison to the leaves of *B. purpurea* (BP) and MEBP

Classes of bioactive compounds	Types of extracts				
	BP	MEBP	PEBP	EABP	AQBP
Alkaloids	-	-	-	-	-
Saponins	+	+	-	+	+
Flavonoids	+	+	++	++	-
Tannins and polyphenolic compounds	+	+	+	+	+
Triterpenes	+++	+	++	-	-
Steroids	+++	++	+++	+++	-

*BP - B. purpurea* leaves, *MEBP* - methanol extract of *B. purpurea*

For saponins - + = 1–2 cm froth; ++ = 2–3 cm froth; +++ = >3 cm froth

For flavonoids, tannins, triterpenes and steroids - + = weak colour; ++ = mild colour; +++ = strong colour

For akalioids - + = negligible amount of precipitate; ++ = mild precipitate; +++ = strong precipitate; – = not detected

### HPLC Profile of PEBP, EABP and AQBP

**Fig. 2:**
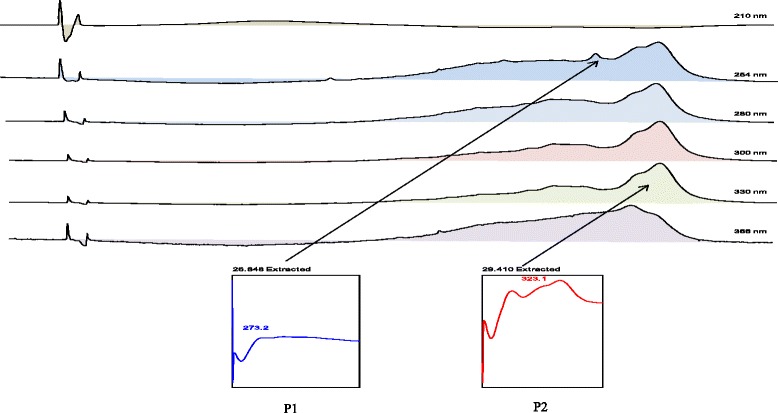
The UV spectra analysis of peak 1 (RT = 26.85 min) and peak 2 (RT = 29.4 min) of the PEBP at 254 nm and 339 nm exhibiting the λ max at 273 nm and 323 nm respectively

The HPLC profiles of PEBP, EABP, and AQBP were measured at the wavelengths of 210, 254, 280, 300, 330, and 366 nm. Clear peak separation was detected at different wavelengths. For PEBP, 2 major peaks were clearly separated at the wavelengths of 254 and 330 nm in the chromatogram at the respective R_T_ of 26.8 and 29.4 min (P1 and P2). The λ_max_ values of the peaks were at 273.2 and 332.1 nm, respectively (Fig. 2).

**Fig. 3:**
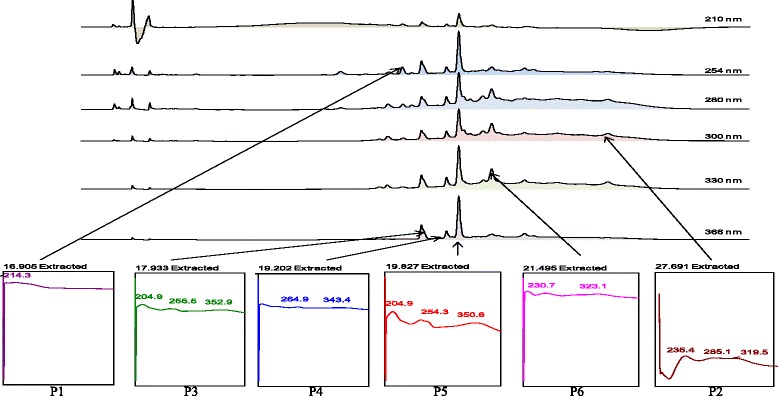
The UV spectra analysis of peak 3 (RT = 17.93 min), peak 4 (RT = 19.20 min) and peak 5 (RT = 19.83 min) of the EABP at 366 nm exhibiting the λ max at 256–353 nm, 265–343 nm and 254–351 nm, respectively, suggesting, in part, the presence of flavonoid-based compounds

The HPLC profile of EABP showed the isolation of 6 major peaks (P1, P2 P3, P4, P5, and P6) at different wavelengths in the chromatogram (Fig. 3) at the respective R_T_ of 16.91 min (254 nm), 27.69 min (300 nm), 21.50 min (330 nm), and 17.93, 19.20, and 19.83 min (366 nm). Further analysis found that all peaks had λ_max_ values in the region of 214.3, 285.1–319.5, 230.7–323.1, 255.5–352.9, 264.9–343.4, and 254.3–350.6 nm, respectively (Fig. 3).

**Fig. 4:**
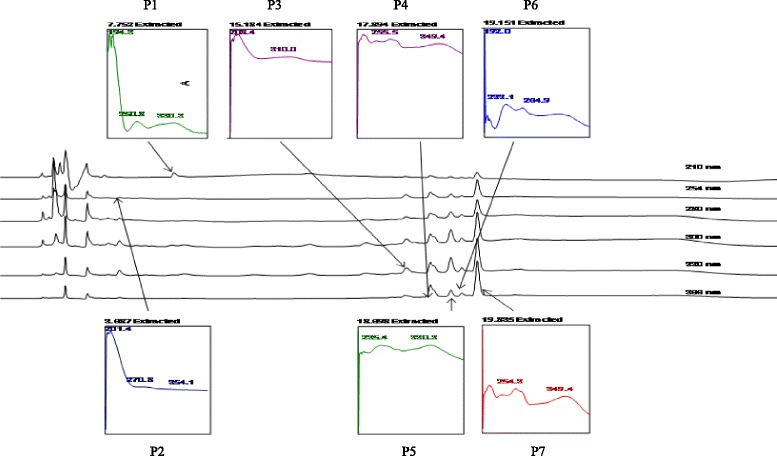
The UV spectra analysis of peak 1 (RT = 7.75 min), peak 2 (RT = 3.69 min), and peak 4 (RT = 17.89 min) and peak 7 (RT = 19.83 min) of the AQBP at 210 nm, 254 nm, and 366 nm respectively. The peaks exhibit the λ _max_ at 251–330 nm, 271–354 nm, 256–349 nm and 254–349 nm, respectively, suggesting, in part, the presence of flavonoid-based compounds

Meanwhile, HPLC of AQBP demonstrated the isolation of 7 peaks at different wavelengths in the chromatogram (P1, P2 P3, P4, P5, P6, and P7) at the respective R_T_ of 7.75 min (210 nm), 3.69 min (254 nm), 15.18 min (330 nm), and 17.90, 18.70, 19.15, and 19.86 min (366 nm), respectively. The peaks were further analyzed, showing λ_max_ values in the region of 250.8–330.3, 270.8–354.1, 208.4–310.0, 255.5–349.4, 235.4–330.3, 233.1–254.9, and 254.3–349.4 nm, respectively (Fig. 4).

## Discussion

PCM is safe when consumed in therapeutic doses; interaction with other drugs and side effects are rarely observed [29]. However, overdose of PCM is known to result in liver injury, with the potential for liver failure progression [30, 31]. Given its clinical relevance, PCM is a widely preferred toxicity model for the evaluation of hepatoprotective agents [29]. Via the glucuronidation process, more than 90 % of PCM that has been consumed in therapeutic doses is conjugated by the liver into inactive components, non-toxic sulphate, and glucuronide prior to their excretion in the urine. Meanwhile, less than 5 % of PCM is metabolized to a highly reactive toxic metabolite: *N*-acetyl-*p*-benzoquinoneimine (NAPQI), via CYP450 [32]. NAPQI is commonly reduced by glutathione (GSH) to a non-toxic mercaptate conjugate. GSH has been highlighted as being responsible for the antioxidant defense in humans by scavenging the free radicals produced through the metabolism processes within the liver [33] in order to prevent subsequent cell damage. Excessive intake of PCM causes the metabolic pathways, particularly the sulphation and glucuronidation pathways, to become saturated, resulting in the shunting of more PCM down the CYP450 pathway to generate unnecessary high amounts of NAPQI. Excessive production of NAPQI depletes the GSH stores in the body [34]. GSH concentrations dropped to low levels in the centrilobular cells, subsequently affecting the ability of GSH peroxidase, the major peroxide detoxification enzyme, to function efficiently under conditions of GSH deficiency. In the situation when the GSH supply decreases to less than 30 % of the normal, the unbound NAPQI will bind nonspecifically to tissue macromolecules, including intracellular proteins, particularly those with sulfhydryl groups, consequently causing cell dysfunction and death [32, 35].

Despite the advancement in the field of modern medicine with the development of various types of new drugs to treat various types of ailments, their effectiveness is sometimes surpassed by their adverse side effects. Therefore, researches are being carried out throughout the world to find alternative drugs with less, or, possibly, no side effects to help treat those ailments. In the case of liver toxicity, limited number of drugs has been developed to help fight this disease, but their uses are usually accompanied by unwanted side effects. In an attempt to contribute to the search for potent and safe liver-protecting agent, the hepatoprotective potential of *B. purpurea* was investigated. The reason for choosing *B. purpurea* was based on the findings that the plant possesses remarkable antioxidant and anti-inflammatory activities [7, 8, 9, 12, 13], which have been generally known to play part in the mechanisms of hepatoprotection.

In the present study, three types of partitions, namely PEBP, EABP and AQBP, were prepared from MEBP and subjected to the PCM-induced liver toxicity in rats’ model. From the results of serum biochemical analysis, EABP was found to be the most effective partition followed by the AQBP with PEBP shows no effect on the liver function enzymes level. Only EABP, at all doses tested,  were able to reduce the serum enzymes’ level, which was earlier increase by the overdose PCM. This finding was supported by the microscopic observations and histopathological scoring. Overall, these observations suggested that the hepatoprotective activity of *B. purpurea* could be due to the synergistic action of a mixture of polar and non-polar compounds with the intermediate polarity compounds being the main hepatoprotective compounds in *B. purpurea*. Moreover, the suggested synergistic action of compounds present in EABP was further supported by the phytochemical analysis of EABP that demonstrated the presence of flavonoids, tannins and saponins. These classes of compounds have been reported to exert antioxidant [36, 37, 38] and anti-inflammatory [39,40, 41] activities.

TPC values were influenced by the non-polar to polar solvent extractions. In this case, the value decreased in the following order: EABP > AQBP > PEBP. Based on the solvent polarity chart, ethyl acetate is classified as an intermediate solvent, hence is able to extract intermediate-polarity phenolic compounds, while aqueous and petroleum ether extract polar and non-polar compounds, respectively. The present findings were further supported by Azlim Almey et al. [42], who suggested that higher extraction yields of total extractable polyphenols and total soluble solids are collected as the solvent polarity increases. In the present study, although EABP was considered to have the highest TPC value (271 mg GAE/100 g extract) in comparison to the other partitions (PEBP = 105 mg GAE/100 g extract; AEBP = 133 mg GAE/100 g extract), the value was lower than the standard value (>1000 mg GAE/100 g extract) for any substances to be considered as having a high TPC [19]. It is important to highlight that MEBP did possess a high TPC value of approximately 1218 mg GAE/100 g extract [14]. Usually, it is anticipated that extracts with a high amount of polyphenolic content should also exhibit high antioxidant activity [43]. However, there is also report on the presence of extract with low TPC value exhibiting high antioxidant activity [44], which supported the presence finding as demonstrated by EABP. Reihani and Azhar [45], while discussing on their finding that the curry leaf demonstrated a comparatively low antioxidant activity despite showing the highest TPC value, cited that the difference could be related to poor specificity of the TPC assay [46, 47]. Moreover, Singleton et al. [47] also suggested that the phenolic compounds, depending on the number of phenolic groups they have, react differently to the Folin–Ciocalteu reagent.

In discussing the relationship between TPC value and antioxidant activity, it is worth to discuss on phenolic compounds in general. Phenolic compounds, broadly distributed in the plant kingdom, are the most abundant secondary metabolites of plants. They are compounds having one or more aromatic rings with one or more hydroxyl groups. Currently, more than 8000 phenolic structures have been identified, ranging from simple molecules (i.e. phenolic acids) to incredibly polymerized substances (i.e. tannins). The most common plant phenolics include phenolics acids, flavonoids, tannins, with flavonoids being the most plentiful polyphenols in our diets. Flavonoids are themselves classified into several subclasses depending on the oxidation state of the central C ring. The five well-known subclasses of flavonoids are flavonols, flavones, flavanones, flavanonols, and dihydroflavonols. Their structural dissimilarity in each subgroup is in part attributable to the degree and pattern of hydroxylation, methoxylation, prenylation, or glycosylation [48]. Tannins, another major group of polyphenols, are usually subdivided into two classes, namely hydrolysable tannins and condensed tannins. Hydrolysable tannins are compounds containing a central core of glucose or another polyol esterified with gallic acid (known as gallotannins), or with hexahydroxydiphenic acid (known as ellagitannins). The high diversity in the structure of these compounds is attributable to the many possibilities in forming oxidative linkage [49]. On the other hand, condensed tannins are oligomers or polymers of flavan-3-ol connected through an interflavan carbon bond. The structure diversity of this class of tannins can be due to various factors such as the discrepancy in hydroxylation pattern, stereochemistry at the three chiral centers, and the location and type of interflavan linkage, as well as the degree and pattern of methoxylation, glycosylation and galloylation [50]. The fact that there are various types of polyphenolic compounds in EABP might support the claim made by Singleton et al. [47] that those polyphenolic compounds react differently to the Foin-Ciocalteu reagent resulting in the low TPC value obtained.

Polyphenolics, as antioxidants, are believed to have the capability to donate hydrogen to free radicals, consequently breaking the chain reaction of lipid peroxidation at the initiation stage [51]. The fact that EABP exerted high antioxidant activity when assess using the superoxide radical scavenging and ORAC assays seems to suggest the presence of high polyphenolic compounds in EABP. However, this suggestion seems to be contradicted by EABP’s low TPC value indicating that the antioxidant activity did not correlate with the TPC value. According to Wong et al. [52], since TPC value is obtained following the Folin–Ciocalteu reaction, which is based on redox reactions, the assay detects not only polyphenolic compounds, but also other biological substances that are reactive towards the Folin–Ciocalteu reagents (i.e. amino acids, carbohydrates and ascorbic acid) [46, 47]. It is plausible to suggest that some of the polyphenolic compounds present in EABP did not work via redox reaction against the Folin-Ciocalteu reagents leading to low TPC value. In addition, the low to moderate antioxidant activity observed in the DPPH radical scavenging assay could be due to the presence of some polyphenolic compounds that may not scavenge DPPH radicals (DPPH●) due to steric resistance [52]. Following the DPPH radical scavenging assay, EABP was also found to exert the highest antioxidant activity further suggesting the parallel relationship between total phenolic value and antioxidant activity. In the SOD scavenging assay, EABP again demonstrated potential free radical scavenging activity. The ability of EABP to effectively attenuate both assays suggests its potential to work via the mechanism of reduction. Interestingly, EABP and AQBP were also able to exert remarkable antioxidant activity when measured using the ORAC assay, which is based on the peroxyl radical absorbance capacity of an extract [53]. Based on all of the above analysis, EABP is believed to have promising antioxidant activity followed by AQBP and PEBP.

Following subjection to the in vitro LOX and XO assays, all partitions of MEBP were found to exert very low anti-inflammatory activity. LOXs are enzymes that have a major role in manipulating the biosynthesis of leukotrienes, which have been ascribed to the pathophysiology of several allergic and inflammatory diseases [54]. Meanwhile, XO is important for catalyzing the sequential oxidation of hypoxanthine to xanthine, and to the production of hydrogen peroxide and uric acid [55]. The enzymatic activity via the LOX and XO pathways essentially involve modulation of the inflammation mechanism [56, 57]. Although the present anti-inflammatory findings might be in contrast to previous anti-inflammatory report on *B. purpurea* [15], several factors are to be considered. Firstly, the type of inflammatory models used to evaluate the anti-inflammatory potential of extract and partitions were significantly different, wherein assessment of the earlier was made using in vivo models whereas assessment of the latter was made using the in vitro models. Moreover, the in vivo models used were more related to the cyclo-oxygenase (COX)-dependent model [15]. COX plays a major role in catalyzing the production of prostaglandin [58]. The role of COX and prostaglandin in the modulation of *B. purpurea* pharmacological action could be justified by the ability of MEBP to exert antiulcer activity. As discussed by Zakaria et al. [9] and Abdul Hisam et al. [59], the aqueous (AEBP) or chloroform (CEBP) extracts of *B. purpurea* exert antiulcer activity via a local and non-specific mechanism known as cytoprotection. It is claimed that cytoprotection take place as a result of the ability of certain compounds to trigger the synthesis of prostaglandin, which consecutively lead to the synthesis of mucus and bicarbonate. Thus, from the results obtained it is plausible to suggest that the anti-inflammatory property of *B. purpurea* that may contribute to the hepatoprotective activity of EABP or AEBP occur via the non-LOX, non-XO-, but COX-mediated pathway.

The presence of hepatoprotective activity in *B. purpurea*, particularly its methanol extract (MEBP) and partitions (EABP and AQBP), could be attributed to the presence of various phytoconstituents in the extract and its partitions. Flavonoids, tannins, saponins and triterpenes have been reported elsewhere to exert various pharmacological activities including hepatoprotective activity [40, 60, 61, 62]. Although the presence of other classes of compounds should not be ignored, the presence of flavonoids in the partition, particularly, will be highlighted in this discussion based on the HPLC findings. The presence of flavonoids in *B. purpurea* has been reported earlier by Yadav and Bhadoria [63], supported the presence of flavonoids in MEBP or its partitions, namely EABP and PEBP, as demonstrated by the phytochemical screening data. This claimed was further strengthen by the recent HPLC findings, which show that the detected peaks fall within the UV-Vis spectra (λ_max_) of flavonoid-type compounds [64]. According to Tsimogiannis et al. [64], flavonoids are categorized into five major subgroups: flavonols, flavones, flavanones, flavanonols, and dihydroflavonols. There are 2 absorbance bands on the UV-Vis spectra for determination of flavonoid types wherein for flavonols or flavones the spectra that fall in the respective region of 350–385 nm or 310–350 nm was labeled band A while for band B the spectra lies in the region of 250–290 nm. EABP had 3 major peaks detected from different wavelengths with λ_max_ at 256–353 nm (Fig. 3), and the 4 major peaks of AQBP were detected  at 251–354 nm (Fig. 4). Referring to the chromatogram of the extracts, the major peaks that fell in the region mentioned by Tsimogiannis et al. [64] are apparently flavonoid-based compounds. The most outstanding properties of almost all groups of flavonoids is how they act as antioxidant [65, 66] and anti-inflammatory [67, 68] agents. The ability of EABP to exert the most effective hepatoprotective activity could be linked partly to the presence of flavonoid-based compounds, which might act as antioxidant and COX-modulated anti-inflammatory agents as discussed above. Other than the types of polyphenolic compounds discussed above, it is also important to highlight on the presence of complex polyphenolic compounds such as those that form dimeric flavonoids or those that form a complex with carbohydrate or protein [69]. Focusing on the leaves of *B. purpurea*, our literature search demonstrated that the bioactive compounds identified from the leaves were not single pure polyphenolic compounds but rather dimeric flavonoids of which one of them were associated with carbohydrates, namely bis [3’,4’-dihydroxy-6-methoxy-7,8-furano-5’,6’-mono methylalloxy]-5-C-5-biflavonyl and (4’-hydroxy-7-methyl 3-C-α-L-rhamnopyranosyl)-5-C-5-(4’-hydroxy-7-methyl-3-C-α-D-glucopyranosyl) bioflavonoid [70]. The presence of a complex polyphenolic compounds might explained why there is no match of HPLC peaks when comparison were made between the HPLC chromatogram of MEBP or EABP against several pure flavonoids (i.e. fisetin, quercetin, rutin, quercitrin, naringenin, genistein, pinostrobin, hesperetin, dihydroquercetin, flavanone, 4’.5’7’-trihydroxy flavanone) (data not shown), thus, suggesting that the flavonoids present in MEBP and EABP are not pure polyphenolic compound(s) but rather a complex polyphenolic compounds as described above.

## Conclusion

Based on the above data, it is concluded that the EABP demonstrated the most effective hepatoprotective activity against the PCM-induced liver intoxication in rats in the order of EABP>AQBP>PEBP. The hepatoprotective mechanism of EABP could be linked to its antioxidant activity, possibly via the radical scavenging activity. However, the antioxidant activity of EABP did not correlate with its polyphenolic content. Although EABP only demonstrated low anti-inflammatory activity via the LOX- and XO-mediated inflammation, the ability of EABP to exert anti-inflammatory activity via COX-modulated pathway should not be disregarded based on the previous reports that *B. purpurea* leaves extracts possessed anti-inflammatory activity against the COX-mediated inflammatory models. The hepatoprotective and antioxidant activities of EABP, which were independent of its low TPC value, could also be linked to the presence of several flavonoid-based bioactive compounds. The presence of flavonoid-based compounds partly in EABP were proposed based on two reasons: i) the analysis of each peak obtained from HPLC study revealed that the wavelength fall within the range that reflects flavonoid-based bioactive compounds, and ii) the phytoconstituents screening of EABP demonstrated in part the presence of flavonoid-based compounds. Moreover, these flavonoids might synergistically act with several saponins and tannins, detected during the  phytocontituents screening, to exert the hepatoprotective and antioxidant activities. These results support the medicinal value of *B. purpurea* in general and may suggest the therapeutic potential of EABP, in particular, for the treatment of inflammatory disease, including liver injury.

### Ethics approval and consent to participate

The study protocol involving the use of animals in the present study was approved by the Animal Care and Use Committee (ACUC), Faculty of Medicine and Health Sciences, UPM (Ethical approval no.: UPM/FPSK/PADS/BR-UUH/00450).

### Consent for publication

Not applicable.

### Availability of data and materials

The supporting materials can be obtained upon request via email to the corresponding author.

## Abbreviations

AAPH: 2,2’-Azobis(2-methylpropionamidine) dihydrochloride
ACUC: Animal Care and Use Committee
AEBP: aqueous extract of *B. purpurea*
ALP: alkaline phosphatase
ALT: alanine aminotransferase
ANOVA: one-way analysis of variance
AQBP: aqueous partition of *B. purpurea*
AST: aspartate aminotransferase
*B. purpurea*: *Bauhinia purpurea*
CCl_4_: carbon tetrachloride
CEBP: chloroform extract of *B. purpurea*
COX: cyclo-oxygenase
CYP450: cytochrome P450
DMSO: dimethyl sulfoxide
DPPH: 2,2-diphenyl-1-picrylhydrazyl
EABP: ethyl acetate partition of *B. purpurea*
EDTA: ethylenediaminetetraacetic acid
GA: gallic acid
GAE: gallic acid equivalent
GSH: glutathione
HPLC: high performance liquid chromatography
IC_50_: inhibitory concentration of 50 % population
LDH: lactate dehydrogenase
LOX: lipooxygenase
MEBP: methanol extract of *B. purpurea*
NAC: N-Acetylcysteine
NAPQI: *N*-acetyl-*p*-benzoquinoneimine
NBT: nitroblue tetrazolium
NDGA: nordihydroguaiaretic acid
OD: optical density
ORAC: oxygen radical absorbance capacity
PCM: paracetamol
PEBP: petroleum ether partition of *B. purpurea*
SEM: standard error of mean
SOD: superoxide dismutase
TP: total protein
TPC: total phenolic content
UPM: Universiti Putra Malaysia
UPM: Universiti Putra Malaysia
UV: ultraviolet
X/XOD: xanthine/xanthine oxidase
XO: xanthine oxidase
